# CAR and TCR form individual signaling synapses and do not cross-activate, however, can co-operate in T cell activation

**DOI:** 10.3389/fimmu.2023.1110482

**Published:** 2023-02-01

**Authors:** Markus Barden, Astrid Holzinger, Lukas Velas, Marianna Mezősi-Csaplár, Árpád Szöőr, György Vereb, Gerhard J. Schütz, Andreas A. Hombach, Hinrich Abken

**Affiliations:** ^1^ Leibniz Institute for Immunotherapy (LIT), Division of Genetic Immunotherapy, University Regensburg, Regensburg, Germany; ^2^ Institute of Applied Physics, TU Wien, Vienna, Austria; ^3^ Department of Biophysics and Cell Biology, Faculty of Medicine, University of Debrecen, Debrecen, Hungary; ^4^ ELKH-DE Cell Biology and Signaling Research Group, Faculty of Medicine, University of Debrecen, Debrecen, Hungary; ^5^ Center for Molecular Medicine Cologne, University of Cologne, Cologne, Germany; ^6^ Department I Internal Medicine, University Hospital Cologne, Cologne, Germany

**Keywords:** immunotherapy, adoptive cell therapy, CAR, TCR, synapse

## Abstract

In engineered T cells the CAR is co-expressed along with the physiological TCR/CD3 complex, both utilizing the same downstream signaling machinery for T cell activation. It is unresolved whether CAR-mediated T cell activation depends on the presence of the TCR and whether CAR and TCR mutually cross-activate upon engaging their respective antigen. Here we demonstrate that the CD3ζ CAR level was independent of the TCR associated CD3ζ and could not replace CD3ζ to rescue the TCR complex in CD3ζ KO T cells. Upon activation, the CAR did not induce phosphorylation of TCR associated CD3ζ and, vice versa, TCR activation did not induce CAR CD3ζ phosphorylation. Consequently, CAR and TCR did not cross-signal to trigger T cell effector functions. On the membrane level, TCR and CAR formed separate synapses upon antigen engagement as revealed by total internal reflection fluorescence (TIRF) and fast AiryScan microscopy. Upon engaging their respective antigen, however, CAR and TCR could co-operate in triggering effector functions through combinatorial signaling allowing logic “AND” gating in target recognition. Data also imply that tonic TCR signaling can support CAR-mediated T cell activation emphasizing the potential relevance of the endogenous TCR for maintaining T cell capacities in the long-term.

## Introduction

Chimeric antigen receptors (CARs) can be remarkably powerful in redirecting a T cell response towards defined target cells (1) while utilizing the TCR/CD3 downstream signaling machinery for triggering T cell activation upon target engagement. Most “second generation” CARs in clinical application incorporate the CD3ζ signaling chain to provide the primary signal together with the costimulatory domain to add the second signal in order to trigger T cell activation (2–5). While this type of CAR is efficacious in clinical application, little is known whether the endogenous TCR/CD3 complex affects the stability and function of the CAR and vice versa. This is a relevant issue since conventional CAR T cells express a functionally active TCR/CD3 complex with the consequence that the CAR competes with the TCR for downstream signaling molecules (6, 7). This situation may result in a functional cross-talk between CAR and TCR upon either target recognition. The issue is also of relevance when replacing the TCR α−chain locus of the endogenous TCR by the CAR encoding DNA sequence (8) thereby producing TCR-deficient CAR T cells. “Off-the-shelf” CAR T cell therapy also uses TCR^-^ T cells for manufacturing (9). In both situations, CAR redirected T cell activation would not compete with the endogenous TCR, however, would not get “help” by tonic TCR signaling.

The TCR associated CD3ζ chain is crucial for regulating the stability of the entire TCR complex and experiences a rapid turn-over on the T cell membrane independently of the other TCR chains (10). The impact of the TCR associated CD3ζ chain on the CD3ζ-based CAR with respect to expression and function was so far not addressed. Mutual co-regulation of the TCR and CAR would have substantial consequences for both CAR- and TCR-mediated T cell activation. This became most recently obvious when CAR T cells with genetically deleted TCR experienced reduced persistence *in vivo* compared to CAR T cells with the endogenous TCR (11). On the other hand, TCR^+^ CAR T cells showed superior persistence implying that the CAR cannot fully substitute for the TCR in sustaining downstream functional capacities.

We asked whether the TCR affects a CD3ζ CAR, and vice versa, in T cell activation on the membrane level of chain phosphorylation and on the downstream level of effector functions. We revealed that TCR and CAR are co-regulated on the T cell surface and can complement in providing downstream T cell activation. However, there is no cross-phosphorylation of CAR and TCR CD3ζ signaling chains. Accordingly, the TCR is not recruited into the CAR synapse as revealed by TIRF and fast AiryScan microscopy. Such lack of cross-signaling allows Boolean logic “AND” gating during combinatorial antigen recognition through the TCR and CAR.

## Materials and methods

### Cell lines and cell culture

The murine T cell hybridoma line MD45 was described elsewhere (12). The human Jurkat T cell line (ATCC TIB-152), the N87 (ATCC CRL-5822) and the CA19−9^+^ and CA19−9^-^ human tumor cell lines LS174T (ATCC CCL-188), H498 (ATCC CCL-254), H716 (ATCC CCL-251) and A375 (ATCC CRL-1619) were obtained from ATCC. Jurkat 76 cells lacking endogenous TCR expression (13) were kindly provided by Dr M.H.M. Heemskerk, Leiden, The Netherlands. Cell lines were cultured in RPMI 1640 medium supplemented with 10% (v/v) heat inactivated FCS. The N87 human gastric carcinoma cell line was cultured in Dulbecco’s Modified Eagle Medium (DMEM) supplemented with 2 mM GlutaMAX, 10% (v/v) FCS and antibiotics. HEK293T cells are human embryonic kidney cells that express the SV40 large T antigen (14). Anti-CD3 mAb OKT3 and anti-CD28 mAb 15E8 were purified by affinity chromatography from supernatants of OKT3 hybridoma (ATCC CRL 8001) and 15E8 hybridoma cells (kindly provided by Dr. R. van Lier, Red Cross Central Blood Bank, Amsterdam, The Netherlands), respectively. The anti-BW431/26 idiotypic antibody BW2064 was described earlier (15). Recombinant ErbB2-Fc protein was purchased from R&D Systems, Wiesbaden, Germany. The PE-conjugated and the AF647-conjugated F(ab´)_2_ goat anti-human IgG antibody, goat anti-human IgG-UNLB antibody, goat anti-mouse IgG human ads-UNLB and rabbit anti-goat IgG (H+L)-UNLB antibody were purchased from Southern Biotechnology, Birmingham, AL, USA. PE-conjugated anti-CD3ζ mAb clone 6B10.2 and AF647-conjugated anti-TCR α/β mAb clone IP26 was purchased from BioLegend, San Diego, CA, USA. Fluorochrome-conjugated anti-human CD3 mAb was purchased from Miltenyi Biotec, Bergisch Gladbach, Germany. Fluorochrome-conjugated isotype controls were purchased from BD Biosciences, San Diego, CA, USA. Matched antibody pairs for capture and detection of human IFN-γ and IL-2 were purchased from BD Biosciences. Recombinant IL-2 was obtained from Endogen, Woburn, MA, USA. Alkaline phosphatase conjugated streptavidin was purchased from Roche Diagnostics, Mannheim, Germany. Peroxidase-labeled goat anti-human IgG Fc antibody and peroxidase-labeled anti-mouse IgG Fc antibody were purchased from Dako, Hamburg, Germany. Anti-actin antibody (clone 1A4) was purchased from Thermo Fisher Scientific, Dreieich, Germany. AF647-conjugated transferrin receptor monoclonal antibody (MEM-75) was purchased from Invitrogen, Regensburg, Germany.

### Genome editing of Jurkat cells

Deletion of CD3ζ in Jurkat cells was performed by CRISPR/Cas9 mediated genome editing utilizing the CD3ζ CRISPR/Cas9 ko plasmid coding for a human CD3ζ guide RNA and the CD3ζ homology directed repair (HDR) plasmid for site specific integration of a puromycin resistance gene (both Santa Cruz Biotechnology, Dallas, TA, USA). Briefly, 5 x 10^6^ Jurkat cells were transfected with 2 µg of each plasmid DNA utilizing the MACSfectin transfection system (Miltenyi Biotec) according to the manufacturer´s recommendations. Two days after transfection cells were further cultured in presence of 250 ng/ml puromycin (Sigma Aldrich, Taufkirchen, Germany). Puromycin resistant subclones were established and tested for expression of CD3ζ by flow cytometry and Western blot analysis.

### Preparation of human T cells

Peripheral blood lymphocytes were obtained from healthy donors by Ficoll density centrifugation (Ethic approval 01-090 Cologne; Ethic approval 21-2224-101 Regensburg). T cells were initially activated by OKT3 (100–200 ng/ml) and 15E8 (50–100 ng/ml) antibodies and IL-2 (400–1,000 U/ml) and further cultured in the presence of IL−2 (100–500 U/ml).

### Engineering and expression of CARs

Cloning and expression of CAR constructs were described previously (5, 16–19). MD45 T hybridoma cells with stable expression of ζ- and γ−chain CARs were generated as follows: The DNA for ζ- and γ−chain CARs in pRSV (50–100 µg) was transfected into 2 x 10^7^ MD45 T cells by electroporation (one pulse, 250 V, 2400 µF) using a gene pulse electroporator (BioRad, Munich, Germany). After culture for two days, transfected cells with CAR expression were selected in the presence of G418 (2 mg/ml; Gibco, Eggenheim, Germany). For expression of CARs in peripheral blood T cells and Jurkat cells all CARs were cloned into the same retroviral expression vector as previously described (20). Transduction of T cells was previously described (5, 20, 21). Briefly, peripheral blood T cells were activated with anti−CD3 (100–200 ng/ml) and anti−CD28 (50–100 ng/ml) antibodies and IL−2 (400–1,000 U/ml). Cells were transduced on day 2–3 by co-cultivation with virus producing 293T cells or, alternatively, with γ-retrovirus containing supernatants. Retroviruses were transiently produced by 293T cells upon transfection with vector DNA and plasmids encoding the GALV envelope and MMLV derived gag/pol (21). For transient expression in non-lymphoid cells, CAR encoding DNAs were transfected in 293T cells. CAR expression was monitored by flow cytometry using an antibody against the common extracellular IgG1 Fc domain.

### Immunofluorescence and flow cytometry

The CAR on the cell surface of engineered T cells was detected by FITC- or PE-labeled antibodies against the human IgG1 Fc domain and T cells were identified with fluorochrome-labeled anti−CD3 antibodies which recognize an epitope located on the ϵ-chain of the CD3 complex, respectively. Flow cytometry was performed using a FACScan™ cytofluorometer equipped with the FACScan™ research software type-B (BD Bioscience), a FACSCanto II flow cytometer equipped with the FACSDiva software (BD Bioscience), and FACSLyric flow cytometer equipped with FACSuite software (BD Bioscience). To monitor expression of the ζ−chain, cells were permeabilized and fixed utilizing the Cytofix/Cytoperm™ reagent kit (BD Bioscience) prior to incubation with the PE-conjugated anti-CD3ζ mAb (2 µg/ml).

### Pulse chase labeling of CARs

CAR engineered cells (5 x 10^7^ cells/ml) were washed twice in cold PBS, pH 7.6, and incubated with 100 µg/ml biotin-ε-amidocaproate-N-hydroxy-succinimidester (Sigma-Aldrich) for 60 min on ice. Cells were washed three times in RPMI 1640 medium, 10% (v/v) FCS, and incubated with or without the anti−IgG antibody (1 µg/ml) at 37°C to cross-link the CAR. Aliquots of cells (10^7^ cells) were spun down at different time points and lysed by adding 100 µl lysis buffer (1% (v/v) NP40, 150 mM NaCl, 50 mM Tris/HCl, pH 8, 10 mM EDTA, 1 mM PMSF, 10 mM iodoacetamide. After 30 min on ice, the lysates were cleared by centrifugation. Nuclei free supernatants (100 µl) were stored at -20°C. Lysates were added to microtiter wells coated with anti−IgG antibody (1 µg/ml) and incubated for 2 h at room temperature. The bound biotinylated CAR was detected by alkaline phosphatase conjugated streptavidin (1:10,000). The reaction product was developed with pNPP (Sigma-Aldrich).

### SDS PAGE and western blot analysis

For analysis of protein half-life on T cell surface, protein synthesis of CAR transfected cells (5 x 10^7^/ml) was blocked by culture in the presence of cycloheximide (10 µg/ml). Cells were lysed (5 x 10^7^) at different time points, lysates separated by SDS-PAGE in 8% (w/v) polyacrylamide gels under non-reducing conditions and subsequently blotted onto a PVDF membrane (Thermo Fisher Scientific). The membrane was probed with the peroxidase-labeled goat anti−human IgG Fc antibody to detect the CAR (1:10,000). For loading control blots were stripped and probed with an anti−actin antibody (0.5 µg/ml) and peroxidase-labeled anti−mouse IgG Fc antibody (1:5,000). Bands were visualized by chemoluminescence utilizing the “ECL Western blotting detection system” (Amersham Biosciences, Freiburg, Germany). Intensity of bands was densitometrically quantified utilizing the ImageJ software. Data were presented as percent of the intensity at time 0. To monitor expression of endogenous CD3ζ chain, lysates of non-modified and CD3ζ genome edited Jurkat cells were separated by SDS-PAGE in 12% (w/v) polyacrylamide gels under reducing conditions, blotted and probed with the anti−CD3ζ mAb (clone 4B10, Thermo Fisher Scientific). Bound antibodies were detected by a peroxidase-conjugated anti−mouse IgG antibody (Sigma Aldrich) at 1:5,000 dilution. Membranes were stripped and re-probed with the peroxidase-labeled anti−β-actin antibody (Santa Cruz Biotechnology) at 1:20,000 dilution. Bands were visualized by chemoluminescence. To monitor expression of phosphorylated CD3ζ, cells were resuspended in RIPA buffer and protein concentrations were determined by ROTI-Quant (Carl Roth, Karlsruhe, Germany). For Western blot analysis, lysates were electrophoresed by SDS-PAGE in 4–12% (w/v) Bis-Tris gels under reducing conditions, blotted and probed with the anti−phospho-CD247 (CD3 zeta) (Tyr142) mAb (clone EM-54, Thermo Fisher Scientific) at 1:1,000 and detected by the peroxidase-labeled anti-mouse IgG1 (γ−chain specific) antibody (Sigma-Aldrich) at 1:10,000 dilution. Membranes were stripped and re-probed with peroxidase-labeled anti−β-actin antibody (Santa Cruz Biotechnology) at 1:20,000 dilution. Bands were visualized by chemoluminescence (ChemiDoc Imaging System, BioRad).

### Total internal reflection fluorescence microscopy

All images were recorded using a home-built setup based on an Olympus IX73 (Japan) microscope body equipped with a high NA objective (Carl Zeiss, alpha-plan apochromat, 1.46 NA, 100x, Germany), 488 nm and 640 nm excitation lasers (OBIS Laser box, Coherent, USA), a quad dichroic mirror (Di01-R405/488/532/635, Semrock, USA) and an emission filter (ZET405/488/532/642m, Chroma, USA). The emission path was split into two color channels using a dichroic mirror (H 643 LPXR superflat, Chroma, USA) and emission filters (650/SP BrightLine HC Shortpass, Semrock, USA; 690/70 H Bandpass, AHF, Germany); the two color channels were imaged onto the same EM-CCD camera (Ixon Ultra, Andor, UK). Prior to measurements, CAR engineered T cells were labeled with either anti−TCRα/β AF647-conjugated full antibody, AF647-conjugated F(ab´)_2_ goat anti−human IgG antibody or AF647-conjugated anti−transferrin receptor (TfR) monoclonal antibody, and seeded on glass slides coated either with recombinant HER2 protein or the anti−CD3 antibody OKT3. Cells were fixed 20 minutes post seeding and imaged by TIRF microscopy upon illumination at 488 nm for CAR-GFP and 640nm for TCR-AF647, for CAR-AF647, or for TfR-AF647. Data analysis was performed with custom Python code (version 3.6) utilizing the following libraries: numpy, mpl_toolkits, scipy, sdt, pandas, matplotlib, seaborn (22–24). The code is available upon request from the corresponding author. Data analysis was performed on regions of interest which included exclusively pixels above a user-defined threshold in at least one of the two color channels. To quantify the size of the contact region, the number of selected pixels was determined and multiplied by the pixel size of160x160 nm^2^. To quantify the extent of CAR and TCR co-localization, the Pearson’s correlation coefficient was calculated *via* $r=\frac{\sum^{}\left(x-\bar{x}\right)\left(y-\bar{y\;}\right)}{\sqrt{\sum^{}{\left(x-\bar{x}\right)}^{2}\;\sum^{}{\left(y-\bar{y}\right)}^{2}}}$ , where *x* and *y* denote the intensity per pixel, and $\bar{x}$ and $\bar{y}$ the corresponding average.

### Fast AiryScan and confocal microscopy

Images were recorded with an LSM 880 confocal laser scanning microscope (Carl Zeiss, Jena, Germany) equipped with an AiryScan/AiryScan Fast detection unit providing up to 120 nm lateral and 350 nm axial resolution (25) and a high NA water immersion objective (C-Apochromat, 1.2 NA, 40x). 488 nm and 633 nm excitation lasers were used to avoid spectral overlap, guided by a 488/543/633 nm triple dichroic mirror. Emission was detected in line switch mode through a 495-560BP/660LP dual band filter. Prior to the experiments CAR-transduced primary human T cells were labelled with either human anti−TCRα/β AF647-conjugated full antibody, AF647-conjugated F(ab´)_2_ goat anti−human IgG antibody or AF647-conjugated anti−transferrin receptor (TfR) monoclonal antibody and seeded on HER2 expressing N87 target cells plated on eight-well tissue culture-treated chambered coverslips (ibidi, Gräfelfing, Germany). Images of live anti-HER2 CAR T cells forming contacts with the tumor target were recorded in AiryScan Fast mode. The chamber was incubated at 37°C during the measurement. 3D images of entire cells were captured by optical sectioning applying 0.23 μm step size along the z-axis. ZEN Black 2.3 software was used to process the acquired raw datasets where Wiener filter deconvolution with 3D reconstruction algorithm and automatic filter strength was applied. ZEN Blue 2.3 software was used to render 3D images for illustrative purposes. Confocal image of each analyzed cell was captured for overall orientation purposes. Differential distribution of CAR-GFP (green) and either CAR-AF647, TCR-AF647, or TfR-AF647 (red) in the synaptic contact region, the extrasynaptic membrane, and the whole cell membrane of AiryScan processed 3D images was quantified based on intensity values in the far red and in the green channels in 3D regions of interest generated using the software ImageJ/Fiji (26) with the 3DSuite plugin (27). 3D Mean Filtering was performed on voxels of 3x3x3 pixel radius (equivalent to 0.30x0.30x0.68 μm radius), then images were segmented based on intensity thresholding to acquire 3D regions of interest. 3D ROIs of the synaptic contact region were generated based on the CAR-GFP signal, and of the extrasynaptic membrane based on either CAR-AF647, TCR-AF647, or TfR-AF647. Each synaptic 3D ROI for all analyzed cells was manually verified based on the extent of the contact region visible in the confocal images to exclude ROIs outside the contact region. Each extrasynaptic 3D ROI for all analyzed cells was manually verified to exclude any non-contacting anti-HER2 CAR T cells in the field of view. Whole cell 3D ROIs were generated by merging the synaptic and extrasynaptic membrane ROIs. Mean intensity of the synaptic contact region, the extrasynaptic membrane, and the whole cell membrane was quantified in both green and red channels. Relative intensity values were generated by dividing the mean intensity values of the synaptic contact region and the extrasynaptic membrane by the mean intensity of the whole cell membrane. Pixel-wise correlation was quantified based on intensity values recorded for CAR-GFP (green) and either CAR-AF647, TCR-AF647, or TfR-AF647 (red) in the synaptic contact region and the extrasynaptic membrane 3D ROIs, including only pixels that were, in at least one of the channels, above the threshold determined as the intersect of intensity histograms from the cell-containing and cell-free areas, i.e. expectedly high cross-correlation of non-labeled areas was excluded from analysis. Pearson’s correlation coefficient was calculated separately for each slice in the 3D images using a custom ImageJ/Fiji plugin. The development of this code will be published separately and will be available at GitHub and the Fiji updater. Average PCC values of all slices for each individual cell were calculated and their mean was plotted with SD as error bars across all cells from at least 3 independent experiments. As an exception to this procedure, for control PCC values, only one AiryScan Fast 2D slice was imaged for each unstimulated cell given the relatively high spatial mobility of unengaged lymphocytes. GraphPad Prism 5 software was used for statistical analysis.

### Activation of CAR engineered Jurkat cells

Microtiter plates were coated with anti−human IgG antibody, that binds to the CAR, and the anti−CD3 antibody OKT3 (5 µg/ml each). CAR engineered or non-modified Jurkat T cells (5 x 10^4^/well) were incubated in coated plates for 48 h and IL−2 in the supernatant was determined by ELISA with a solid phase anti−human IL−2 (2 µg/ml) capture and a biotinylated anti−human IL−2 detection antibody (0.5 µg/ml) (BD Bioscience). The reaction product was visualized with a peroxidase-streptavidin-conjugate (1:10,000) and ABTS (Roche Diagnostics).

### Activation of CAR T cells

CAR T cells (0.32 x 10^4^–5 x 10^4^ cells/well) were co-cultivated for 24–48 h in 96-well round bottom plates with tumor cells (2–5 x 10^4^ cells/well). Supernatants were removed and tested for IFN−γ as described below. Specific target cell lysis of CAR T cells was determined by XTT assay as previously described (28). Viability of target cells without T cells was calculated as the OD-mean of six wells containing only tumor cells subtracted by the background OD-mean of wells with medium only. Non-specific formation of formazan by T cells was determined from ODs of triplicate wells containing exclusively T cells and in same numbers as in the corresponding experimental wells. Viability of target cells in experimental wells was calculated by: viability (%) = [OD(experimental wells - corresponding number of T cells)]/[OD(tumor cells only - medium)] x 100. Cytotoxicity (%) was calculated by: cytotoxicity (%) = 100 - viability (%). Alternatively, CAR T cells (2.5–5 x 10^4^ cells/well) were incubated in 96 well microwell plates coated with the agonistic anti−CD3 (1 µg/ml), anti−CD28 (5 µg/ml), anti−TCR (4 µg/ml), anti−IgG (1 µg/ml) antibodies, anti−idiotypic antibody (BW2064/36; 8 µg/ml) or recombinant HER2-Fc protein (8 µg/ml), respectively. After 48 h supernatants were tested for IFN−γ and IL−2 by ELISA utilizing solid phase bound anti−IFN−γ and anti−IL−2 capture antibodies (each 1 µg/ml) and biotinylated anti−IFN−γ (0.5 µg/ml) and anti−IL−2 detection antibodies (1 µg/ml), respectively. The reaction product was visualized as described above.

## Results

**Figure 1:**
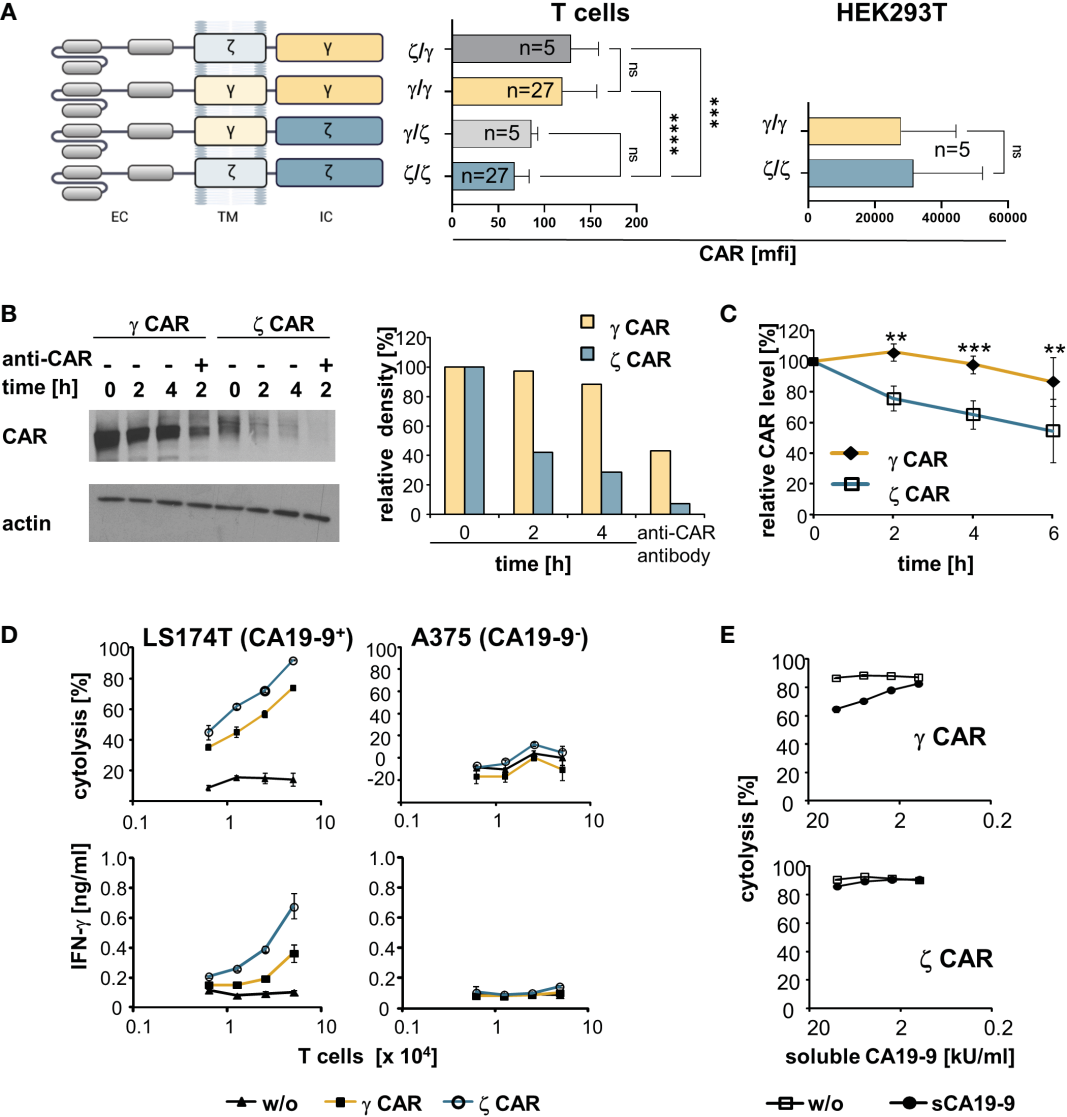
CARs with an intracellular CD3ζ chain are superior over γ−chain CARs in T cell activation despite lower cell surface expression and shorter half-life on the T cell surface. **(A)** Schematic representation of CARs with their respective transmembrane (TM) and intracellular (IC) signaling domains consisting of the respective CD3ζ (ζ) or the FcεRI γ (γ) chain. All CARs harbour the same extracellular domain (EC) and were expressed by the same promoter in the same retroviral vector backbone. Cells were engineered with the respective CARs. CAR expression was monitored by an anti−IgG Fc antibody that detects the common extracellular CAR IgG1-Fc spacer domain. Background staining was determined by an isotype control antibody. Data represent the mean values of mean fluorescence intensity (mfi) ± SD. **(B)** MD45 cells were engineered with a ζ or a γ CAR, respectively, and incubated with cycloheximide (10 µg/ml) to block protein synthesis. For comparison, the CARs were additionally cross-linked by an anti−IgG (anti−CAR) antibody, that binds to the extracellular CAR spacer domain, for 2 h to induce CAR internalization. At different time points, 10^7^ cells were lysed and proteins separated by SDS-PAGE on 8% (w/v) polyacrylamide gels under non-reducing conditions. CARs were detected by the anti−IgG Fc-POD antibody (1:10,000), actin was detected by the anti−actin antibody (0.5 µg/ml). Relative density of CAR bands was quantified utilizing the ImageJ software 1.48 and presented as percent of the initial amount at t=0. Data from a representative experiment out of three are shown. **(C)** Pulse-chase CAR labeling. CAR transfected cells were surface-labeled with biotin as described in Materials and Methods, washed and stimulated at 37°C by an anti−IgG antibody (1 µg/ml) directed against the IgG extracellular CAR domain. Aliquots of cells (5 x 10^6^ cells) were lysed at different time points and lysates were subjected to ELISA plates coated with an anti−IgG1 mAb (1 µg/ml) to capture the CAR. Bound labeled CARs were detected by streptavidin POD and visualized with ABTS. OD at time point 0 was set at 100% and relative ODs at indicated time points were calculated. Numbers represent the mean values of three independent experiments ± SD. **(D)** CAR redirected T cell activation. T cells with ζ- or γ−chain anti−CA19−9 CAR were expanded in the presence of IL−2 and co-cultivated (0.625–5 x 10^4^ cells/well) for 48 h with CA19−9^+^ LS174T or CA19−9^-^ A375 tumor cells (5 x 10^4^ cells/well). Supernatants were analyzed for IFN−γ by ELISA, target cell lysis was determined by the XTT assay. Data represent mean values ± SD of two independent experiments. w/o, without CAR. **(E)** Activation of CAR T cells in the presence of soluble CA19−9 antigen. Anti−CA19−9 CAR T cells (5 x 10^4^ cells/well) were co-cultivated for 48 h with CA19−9^+^ LS174T cells (5 x 10^4^ cells/well) in the presence of serial dilutions of supernatants of H498 tumor cells containing about 20,000 U/ml of soluble CA19−9 (sCA19−9). Target cell lysis was determined by the XTT assay. For control, cells were co-cultivated in the presence of supernatants of the CA19−9^-^ cell line H716 lacking soluble CA19−9 (w/o). Data represent mean values ± SD of technical triplicates. For comparison of two groups, significant differences were determined by Student´s T test. For comparisons of three or more groups, one-way ANOVA with Tukey’s *post hoc* test was used. p-values < 0.05 were considered statistically significant (**p<0.01; ***p<0.001; ****p<0.0001; ns, not significant).

Physiologically, the TCR associated CD3ζ chain rapidly recycles on the cell membrane independently of the other TCR components (10). We asked whether a CD3ζ chain CAR is subjected to the same rapid turn-over and tested a set of CARs with the CD3ζ or the FcϵRI γ signaling chain (**Figure 1A**); the other domains of the respective CARs were the same; the CARs were expressed by the same vector. The ζ−chain CARs were consistently present at lower levels on the T cell surface compared to the corresponding γ−chain CARs. Exchange of the intracellular ζ- and γ−chains reciprocally altered the CAR levels on the cell membrane, while exchange of the transmembrane domains did not, indicating that the different CAR levels on the T cell surface were due to the intracellular moiety. For comparison, the γ- and ζ−chain CARs were present at equal levels in non-T cells like HEK293T cells (**Figure 1A**) indicating that the different CAR levels are due to their expression in T cells, most likely due to the presence of the endogenous TCR/CD3 complex.

We addressed whether the different ζ- and γ−chain CAR levels go along with different protein half-life times on the T cell surface. Blocking protein synthesis and Western blot analyses revealed that the ζ−chain CAR had a shorter half-life time than the γ−chain CAR in engineered MD45 T cells (**Figure 1B**). CAR cross-linking by an anti−IgG antibody, that binds to the common extracellular CAR domain, resulted in rapid degradation of both γ- and ζ−chain CARs as expected. This goes in line with a recent study showing that antigen encounter results in rapid ubiquitination and, as a consequence of internalization and lysosomal degradation, downregulation of CARs (29). Pulse-chase analysis revealed that the ζ−chain CAR molecules on the cell surface more rapidly declined than the γ−chain CARs (**Figure 1C**). Taken together, the ζ−chain CAR experienced a higher turnover on the T cell membrane and a shorter half-life time than the γ−chain CAR.

To record CAR-driven T cell effector functions, peripheral blood T cells were engineered with ζ- and γ−chain CARs with specificity for CA19−9. Recording the cytotoxic activity against CA19−9^+^ and CA19−9^-^ cancer cells revealed that the ζ−chain CAR induced higher cytolytic activity and higher IFN−γ release than the γ−chain CAR indicating a higher potency of the ζ−chain CAR in T cell activation (**Figure 1D**). This was the case despite a lower expression level and lower half-life time compared to the γ−chain CAR on the T cell surface.

Half-life time and rapid turn-over may affect T cell activation in the presence of soluble target antigen. This is a clinically relevant scenario since a substantial number of CAR-targetable surface antigens are also shed by cancer cells which may block the CAR redirected T cell activation. To address the issue, we engineered T cells with the ζ- and γ−chain CAR, respectively, with the same anti−CA19−9 binding domain and co-incubated CAR T cells with CA19−9^+^ target cells in the presence of increasing concentrations of soluble CA19−9 (**Figure 1E**). The induction of lytic activity triggered by the γ−chain CAR was blocked by soluble CA19−9 whereas the activity by the ζ−chain CAR was less affected. We assumed that the rapid turn-over and shorter half-life of the ζ−chain CAR goes along with a rapid replacement by antigen-free CAR chains on surface and thereby a higher resistance towards blocking by soluble antigen.

**Figure 2:**
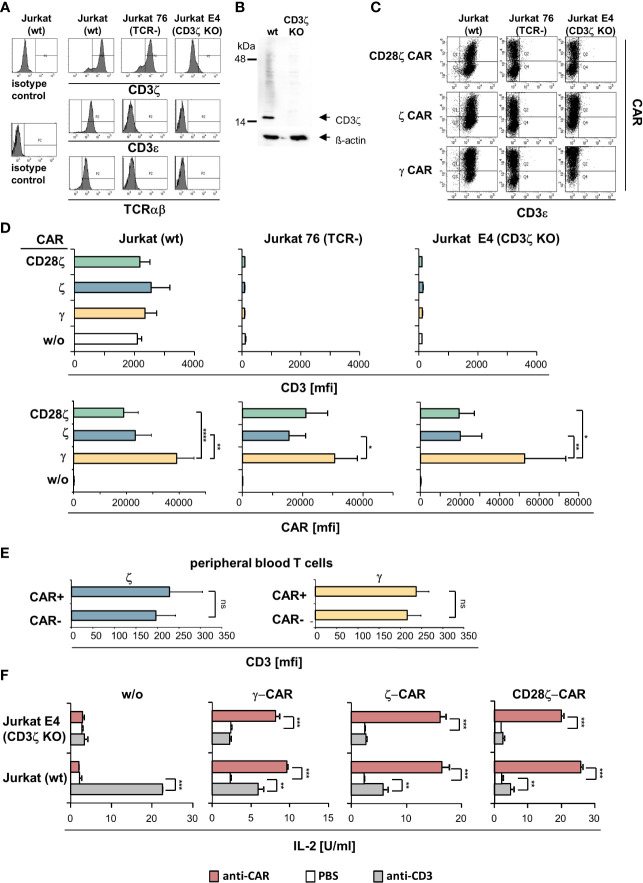
ζ CARs did not rescue the CD3/TCR complex in CD3ζ KO Jurkat cells. **(A)** The CD3ζ locus in Jurkat cells was deleted by CRISPR/Cas9 engineering as described in Materials and Methods. Non-modified Jurkat cells (wt), Jurkat76 cells lacking TCR (TCR^-^) and Jurkat E4 CD3ζ knock-out (KO) cells were tested by flow cytometry for intracellular CD3ζ expression and for surface expression of CD3ε and TCR, respectively. Histograms of a representative analysis are shown. **(B)** Western blots of genome edited Jurkat cells. Lysates of non-modified (wt) and CD3ζ KO Jurkat cells (5 µg protein lysate/lane) were separated by SDS PAGE, blotted, probed with a mouse anti−human CD3ζ antibody (1:500) and detected by a HRP-conjugated anti−mouse antibody (1:5,000). Blots were re-probed with an anti−β-actin antibody (1:20,000). **(C)** TCR^+^ Jurkat (wt), Jurkat76 (TCR^-^) and Jurkat E4 (CD3ζ KO) cells were engineered with the CD28ζ, ζ or γ CAR, respectively. Expression of CARs and surface expression of CD3 was recorded by flow cytometry and mean fluorescence intensity (mfi) was determined. Dot plots of a typical experiment and mean values of 5 independent experiments ± SD **(D)** are shown. Significant differences were determined by Student´s T test. **(E)** Peripheral blood T cells engineered with ζ−chain and γ−chain CAR, respectively, were stained for CAR and CD3 expression and analyzed by flow cytometry. CAR^+^ and CAR^-^ T cells were gated and mean fluorescence intensity (mfi) of CD3 was determined. Data represent mean values of 4 healthy donors ± SD. Statistical differences were determined by Student´s T test. **(F)** Jurkat (wt) and Jurkat E4 (CD3ζ KO) cells with and without CAR, respectively, were stimulated through the CAR and CD3 by incubation on 96-well plates (4 x 10^4^ cells/well) coated with the agonistic anti−CD3 antibody OKT3 or anti−IgG Fc antibody (5 µg/ml each) that binds to the CAR extracellular domain. After 48 h supernatants were tested for IL−2 by ELISA. Values represent the means of technical triplicates ± SD. Significant differences were determined by Student´s T test. A representative experiment out of two is shown. p-values <0.05 were considered statistically significant (*p<0.05; **p<0.01; ***p<0.001; ****p<0.001; ns, not significant).

We asked whether a ζ−chain CAR can substitute for the CD3ζ chain in reconstituting the endogenous TCR, and whether the endogenous CD3 of the TCR affects the expression level of the CD3ζ CAR independently of the TCR αβ chains. To address the issue, we deleted the endogenous CD3ζ chain of Jurkat cells by CRISPR/Cas9 mediated gene editing. Flow cytometry and Western blot analysis demonstrated efficient knockout of the endogenous CD3ζ in engineered Jurkat cells; consequently, no TCR was expressed (**Figures 2A, B**). For comparison, TCR deficient Jurkat76 cells express the endogenous CD3ζ chain as reported (13).

We engineered Jurkat cells without endogenous CD3ζ and/or CD3/TCR expression, respectively, with ζ- and γ−chain CARs (**Figure 2C**). While the CARs were properly expressed by Jurkat cells, the CARs did not rescue TCR expression in CD3ζ KO Jurkat cells (**Figure 2D**). Expression of the endogenous CD3 was not altered by the respective CARs in Jurkat cells. Same data were obtained upon engineering blood T cells (**Figure 2E**). More importantly, ζ−chain CARs were expressed at lower levels in both TCR^-^ CD3ζ^+^ Jurkat76 cells and in TCR^-^ CD3ζ^-^ Jurkat E4 cells compared to the γ−chain CAR indicating that the lower levels of ζ-CARs on T cell surface did not depend on the presence of the TCR or TCR/CD3 complex. With respect to CAR triggered functionality, the ζ- and γ−chain CARs were as active in CD3ζ KO cells as in TCR^+^ CD3ζ^+^ Jurkat cells indicated by cytokine release upon CAR stimulation (**Figure 2F**). For comparison, engineered CD3ζ KO Jurkat cells did not respond upon CD3 stimulation despite the presence of the ζ−chain CAR.

Taken together we concluded that, firstly, the CAR and the CD3/TCR complex are independently regulated on the membrane surface and that the ζ- and γ−chain CARs function independently of the presence of the endogenous CD3/TCR complex in T cells. Secondly, the ζ−chain CAR did not replace CD3ζ in rescuing TCR expression in CD3ζ KO cells.

**Figure 3:**
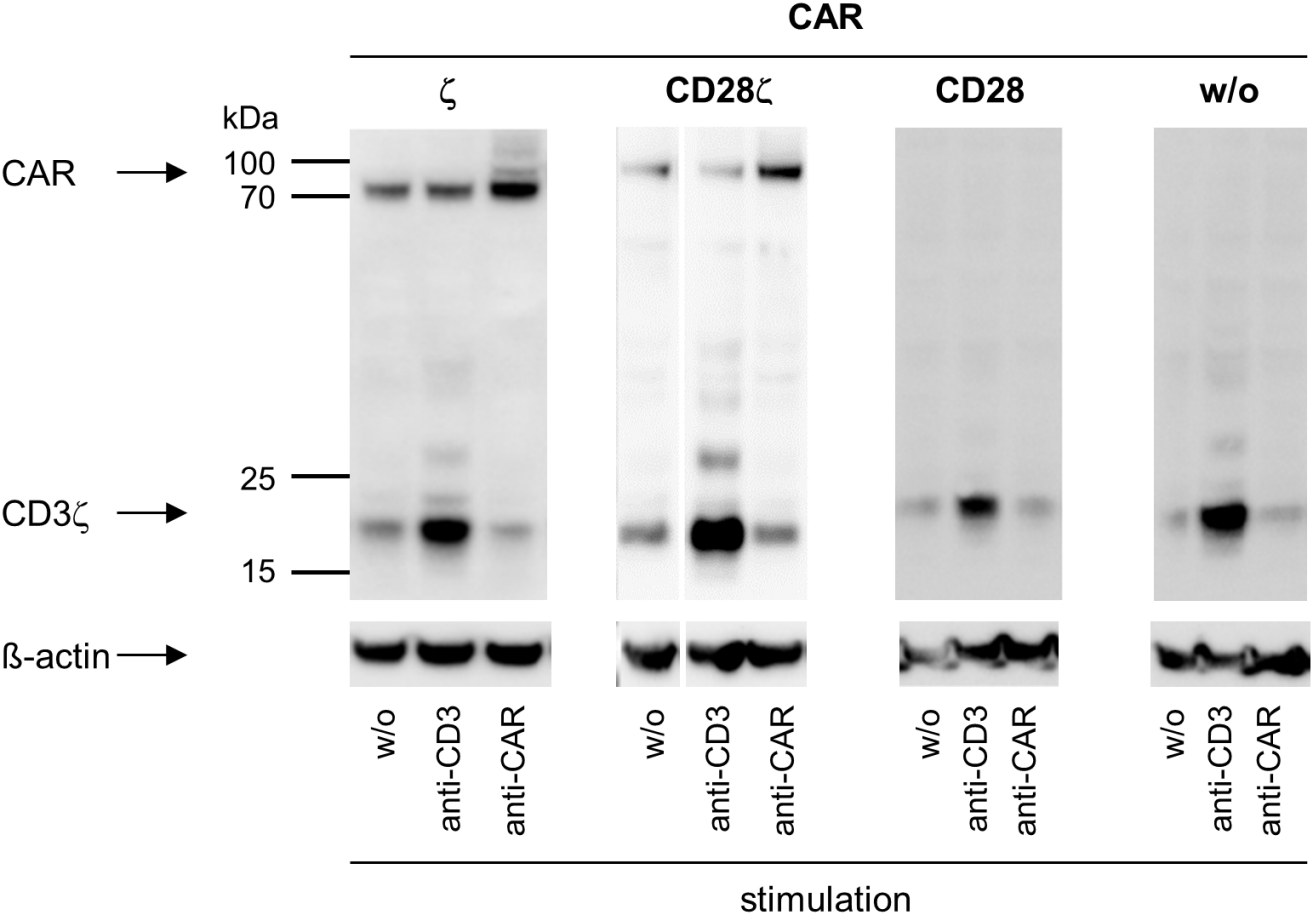
ζ CARs and TCR/CD3 do not cross-activate through their CD3ζ chains. CAR engineered Jurkat cells were recorded for CD3ζ phosphorylation by Western blot analysis. Non-modified (w/o) Jurkat cells or Jurkat cells engineered with ζ CAR, CD28 CAR or CD28ζ CAR (5 x 10^6^ cells each) were subjected stimulation through their TCR by incubation with the agonistic mouse anti−CD3 antibody OKT3 (10 μg/ml) for 10 min followed by an anti−mouse IgG antibody (10 μg/ml) for cross-linking for 3 min (ζ CAR, CD28 CAR and w/o CAR) or 1 min (CD28ζ CAR). Alternatively, cells were stimulated through the CAR independently of the binding domain by incubation with a goat anti−human IgG antibody (10 μg/ml) for 10 min followed by an anti−goat IgG antibody (10 μg/ml) for cross-linking for 3 min (ζ CAR, CD28 CAR and w/o CAR) or 1 min (CD28ζ CAR). Lysates were separated by SDS PAGE and blotted membranes were probed with the anti−phospho-CD247 (CD3ζ) (Tyr142) antibody (clone EM-54) (1:1,000) followed by a peroxidase-conjugated anti−mouse IgG1 antibody (1:10,000) for detection. Blots were re-probed with an anti−human β-actin antibody (1:20,000).

To address whether there is a cross-signaling at the very early stage of TCR and CAR mediated activation, we recorded by Western blot analysis the phosphorylated CD3ζ (pCD3ζ) of the CAR and of TCR-associated, endogenous CD3ζ chain on stimulation. TCR stimulation increased phosphorylation of the TCR associated endogenous CD3ζ chain, but not of the CAR CD3ζ domain (**Figure 3**). Vice versa, stimulation of the CAR resulted in an increased phosphorylation of the CAR CD3ζ chain but not of the TCR CD3ζ chain. The same pattern was obtained with CD28-CD3ζ CAR engineered cells; phosphorylation of CAR CD3ζ increased upon CAR stimulation but not upon TCR/CD3 stimulation; pCD3ζ of the TCR, but not of the CAR, increased upon TCR/CD3 stimulation. We also investigated whether CAR-associated CD28 signaling can induce CD3ζ signaling through the TCR. Stimulation of the CD28 CAR, that lacks the CD3ζ domain, did not produce TCR CD3ζ phosphorylation; increase in pCD3ζ occurred upon TCR/CD3 stimulation as control (**Figure 3**). We concluded that CD28-CD3ζ CAR stimulation did not induce phosphorylation of the endogenous TCR CD3ζ chain indicating that no substantial cross-signaling between the TCR/CD3 and the CAR at the stage of CD3ζ phosphorylation occurred.

**Figure 4:**
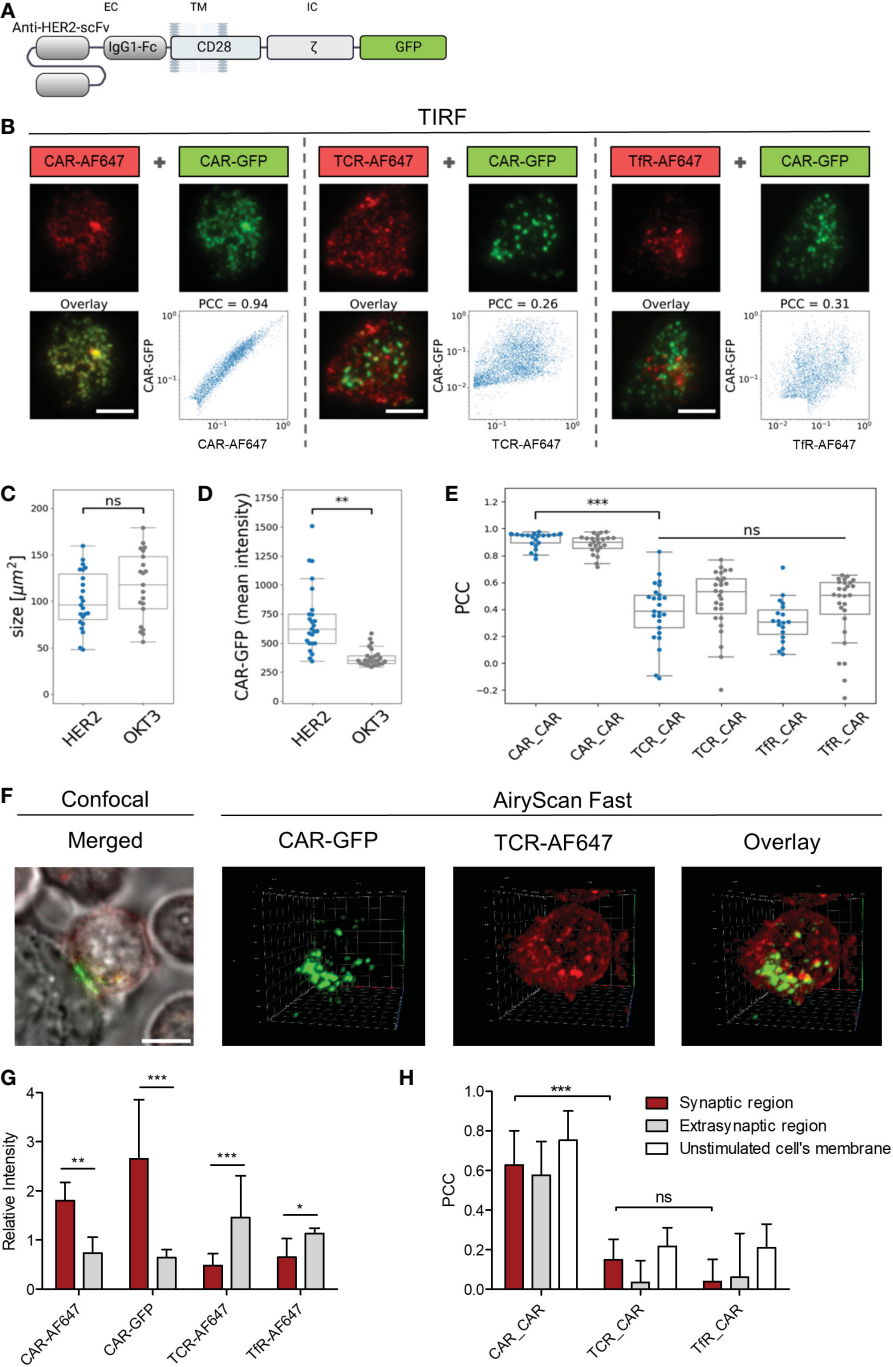
ζ CAR engagement of antigen induced clustering without TCR integration. Anti−HER2 CAR T cells were labeled with either AF647−conjugated F(ab´)_2_ goat anti−human IgG antibody that recognizes the CAR, anti−TCRα/β AF647-conjugated antibody, or AF647-conjugated anti−transferrin receptor (TfR) antibody. **(A)** Schematic representation of the used anti−HER2 CD28ζ CAR linked to GFP at the intracellular site (IC). **(B)** Cells were seeded onto surfaces coated with HER2 protein or anti−CD3 antibody OKT3 and contacts were formed for 20 minutes. Cells were fixed and TIRF microscopy was used to image the localization of CAR-GFP (green) and either CAR-AF647, TCR-AF647, or TfR-AF647 (red). Scale bar represents 5 µm. For each cell, the pixel-wise correlation of the brightness values recorded in the red and in the green channel were plotted along the x- and the y-axis, respectively. Pearson’s correlation coefficient (PCC) for each cell was calculated. Contact size **(C)** and Mean intensity **(D)** of anti−HER2 CAR-GFP plated on HER2 (blue) and OKT3 coated slides (grey) are displayed for n≥16 cells per group in a Whisker box plot. Statistical differences were determined by Wilcoxon-Mann-Whitney test with Python code (**p<0.01). **(E)** PCC for CAR-GFP and TCR-AF647 was calculated for anti−HER2 CAR T cells plated on HER2 (blue) and OKT3 (grey) coated slides and displayed for n≥24 cells per group in a Whisker box plot. Statistical differences were determined by Kruskal-Wallis test with Dunn’s post-hoc test performed with Python code (***p<0.001). **(F)** Labelled anti-HER2 CAR T cells were seeded on HER2 expressing N87 target cells plated on chambered coverslips. 3D fluorescence images of live anti-HER2 CAR T cells forming contacts with the tumor target were recorded in AiryScan Fast mode. Confocal images of each analyzed cell were recorded as controls. **(G)** Differential 3D distribution of CAR-GFP and either CAR-AF647, TCR-AF647, or TfR-AF647 in the synaptic contact region and the extrasynaptic membrane was normalized to total intensity (n_CAR-GFP_=11, n_TCR-AF647 =_ 11, n_CAR-AF647 =_ 10, n_TfR-AF647 =_ 6; 3D images contained approximately 50-80 slices). **(H)** PCC for CAR-GFP and either CAR-AF647, TCR-AF647, or TfR-AF647 in the synaptic contact region and the extrasynaptic membrane was quantified separately for each slice and averaged for each individual cell and presented as the mean of multiple cells across 3 independent experiments. 2D AiryScan Fast images of unstimulated cells were used as control (n_CAR_CAR_=15, n_TCR_CAR_=7, n_TfR_CAR_=14). Data are presented as mean ± SD (*p<0.05, **p<0.01, ***p<0.001; ns, not significant).

To investigate whether CAR and TCR are recruited into similar regions during immunological synapse formation, we engineered peripheral blood T cells with Her2-specific CD28-CD3ζ CARs linked to GFP (**Figure 4A**). The distribution of CAR and TCR in the contact region between CAR T cell and immobilized Her2 molecules was recorded *via* TIRF microscopy. The CAR was localized by its linked GFP and verified by staining with an AF647-conjugated anti−CAR antibody; the TCR was localized by an anti−TCRαβ AF647-conjugated antibody; the transferrin receptor (TfR) was localized by an anti-TfR AF647-conjugated antibody (**Figure 4B**). There was no difference in size of the contact regions formed by the T cell on surfaces coated either with Her2 as CAR target or with the anti−CD3 antibody OKT3 as TCR target (**Figure 4C**). While there was no indication for synapse formation of anti−Her2 CARs on the OKT3 antibody coated surface, recognition of the cognate antigen Her2 led to an accumulation of anti−Her2 CARs in the contact region, but not of the TCR (**Figure 4D**). Notably, the Pearson’s correlation coefficient (PCC) between CAR and TCR distribution was not different compared to the negative distribution (**Figure 4E**, median PCC = 0.384). As negative control, the TfR, that is distributed on the cell surface independently of the CAR and TCR, showed no substantial correlation with the CAR distribution (median PCC = 0.306). As positive control, the GFP-CAR signal strongly correlated with the signal of anti−CAR antibody (median PCC = 0.949).

The distribution of CAR and TCR in the contact region between anti-Her2 CAR T cell and Her2^+^ tumor cell was studied by 3D fast AiryScan microscopy (**Figure 4F**; **Supplementary Figure 1A**; **Supplementary Video 1**, **2**, **3**). The analysis confirmed accumulation of the anti−Her2 CAR, but not of the TCR, in the synaptic region (**Figure 4G**). In fact, TCR was present at lower mean intensities in the synaptic than in the extra-synaptic regions (**Supplementary Figure 1B**), making up a significant difference after normalization to the entire membrane intensity. In anti-Her2 CAR T cells engaging Her2^+^ tumor cells, PCC between CAR and TCR in the synapse was not different from the TfR negative control (mean PCC_CAR_TCR_ = 0.149; PCC_CAR_TfR_ = 0.040), while the GFP-CAR signal showed a strong correlation with the anti−CAR antibody as positive control signal (mean PCC_CAR_CAR_ = 0.628) (**Figure 4H**). No co-distribution of the CAR with the TCR or the TfR as control occurred when the synaptic region, the extra-synaptic region, and the unstimulated CAR T cell membranes were compared (**Figure 4H**). Taken together, data indicate that the CAR synapse formed upon engagement of cognate antigen did not recruit the TCR into the same region.

**Figure 5:**
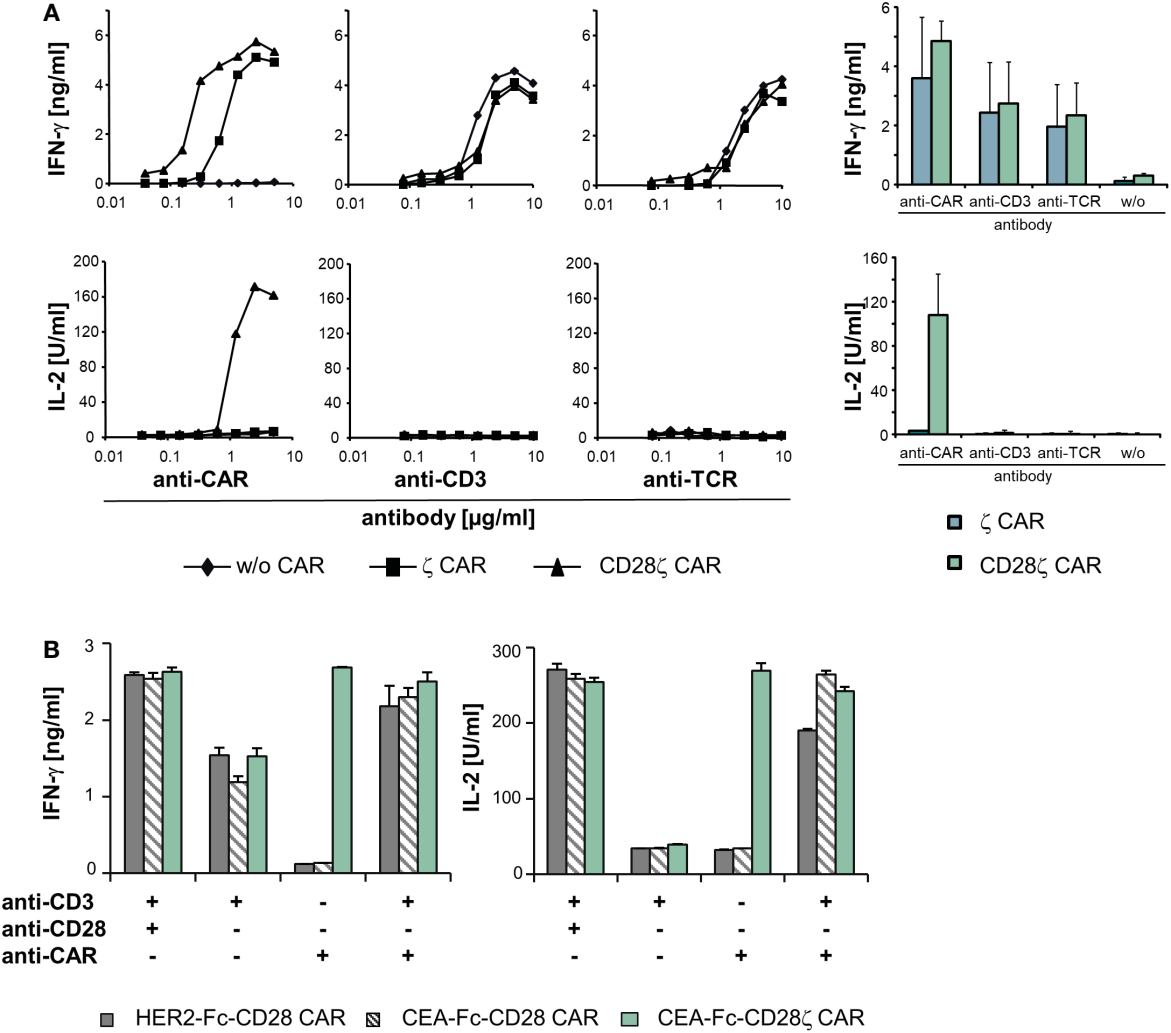
The TCR/CD3 complex and ζ−chain CARs can complement in signaling. **(A)** T cells of healthy donors (5 x 10^4^ cells/well) were engineered with a ζ or CD28ζ CAR, respectively, and cultivated for 48 h in micro-titer plates that were coated with serial dilutions (starting from 10 µg/ml) of an anti−IgG1 Fc antibody for CAR activation or an agonistic anti−CD3 and anti−TCR antibody, respectively. Data from a representative T cell donor are shown; data from four donors were accumulated in mean values ± SD. **(B)** T cells (5 x 10^4^ cells/well) expressing an anti-HER2-Fc-CD28, anti-CEA-Fc-CD28 or anti-CEA-Fc-CD28ζ CAR were cultivated for 48 h in micro-titer plates coated with an agonistic anti−CD3 (1 µg/ml) and anti−CD28 (5 µg/ml) antibody, respectively, or the anti−idiotypic antibody BW2064/36 (8 μg/ml), directed against the binding domain of the anti−CEA CAR, or recombinant HER2-Fc protein (8 μg/ml) recognized by the anti−HER2 CAR, respectively. Combinations of antibodies and/or antigen were used as indicated. Culture supernatants were analyzed for IFN−γ or IL−2 by ELISA as indicated. Numbers represent mean values of technical triplicates ± SD. A representative experiment out of at least three experiments is shown.

As cross-signaling between the CAR and TCR/CD3 can occur at more downstream steps in the activation pathway at the level of effector functions, we recorded cytokine production as a near final step in the activation of effector functions. CAR engineered T cells were stimulated through the CAR, CD3 and TCR, respectively, and IFN−γ and IL−2 release was recorded. The threshold for IFN−γ release by TCR and CD3 activation, respectively, was not altered by the presence of a CD3ζ or CD28-CD3ζ CAR compared to unmodified T cells (**Figure 5A**). While CD3ζ−chain signaling by the CAR was sufficient for IFN−γ secretion, IL−2 release required additional CD28 co-stimulation as provided through the CD28-CD3ζ CAR as expected. No IL−2 release occurred upon TCR or CD3 stimulation in the presence of the CD28-CD3ζ CAR indicating that the co-expressed CD28 CAR domain was not cross-activated by TCR stimulation to complement for IL−2 release.

While TCR and CAR did not cross-activate upon engagement of either cognate antigen, we assessed whether CAR and TCR can complement in T cell activation when both are engaging their respective target. T cells were engineered with a CD28 CAR lacking the CD3ζ domain and recognizing CEA or HER2, respectively. CAR T cells were stimulated through the CAR by binding to their cognate antigen or through their TCR/CD3 (**Figure 5B**). Simultaneous binding to the respective CAR ligand and to an agonistic anti−CD3 antibody induced IL−2 release indicating successful complementation of the TCR/CD3 signaling with CAR CD28 signaling; IL−2 release was not obtained upon TCR/CD3 or CAR stimulation alone. For control, the CD28-CD3ζ CAR induced IL−2 upon binding to the CAR ligand without additional TCR stimulation; stimulation of CD3 plus CD28 independently of the CAR also induced IL−2 release. Data indicate that CAR and TCR/CD3 could complement in the downstream T cell activation pathway when engaging their respective cognate ligand.

## Discussion

Nearly all CARs used in clinical trials signal through the CD3ζ chain by engaging downstream signaling proteins associated with the endogenous TCR/CD3 complex (6, 7). The impact of TCR/CD3 on the CAR redirected T cell activation and vice versa was so far not addressed. A mutual functional interaction is a relevant issue since CAR engineered T cells harbor in addition a functionally active TCR that may interfere with or add to CAR-mediated signaling.

Physiologically, the endogenous CD3ζ stabilizes the CD3/TCR complex; in the absence of CD3ζ, the levels of TCRαβ chains are substantially reduced (30, 31). Moreover, CD3ζ has a rapid turnover on the cell membrane independently of the other TCR components (10). Here we revealed that CD3ζ−chain CARs likewise have a shorter half-life and are expressed at lower levels on the T cell membrane than the FcεRI γ−chain CARs. The low expression levels are mediated by the CAR intracellular CD3ζ and not by the transmembrane domain; the effect holds also true for the second generation CD28-CD3ζ CAR. In contrast to the situation in T cells, γ−chain CARs are less expressed in Fc receptor expressing cells compared with the ζ−chain CARs, like macrophages and neutrophils (32–36); in non-lymphoid cells both γ- and ζ−chain CARs are expressed at similar levels. In CD3ζ KO and in TCR^-^ Jurkat cells the ζ−chain CARs were also less expressed than the γ−chain CARs indicating that the levels of ζ−chain CARs on the T cell surface are affected by downstream elements of the TCR/CD3 complex and not by the presence of the TCR and CD3ζ themselves. Notably, the expression level does not correlate with the activation capacity since CD3ζ CARs require less amounts of antigen than γ−chain CARs to activate engineered T cells.

At the membrane receptor level, the CAR does not co-recruit the TCR into its synapse as revealed by TIRF and fast AiryScan microscopy; vice versa, the TCR does not recruit the CAR into its synapse (37). Consequently, CAR and TCR do not cross-signal with respect to CD3ζ phosphorylation; TCR/CD3 stimulation did not result in increase in CAR CD3ζ phosphorylation and, conversely, CAR stimulation did not increase TCR/CD3 phosphorylation. The conclusion holds true for both the CD3ζ and the CD28-CD3ζ CAR. In line with this finding, costimulatory CD28 signaling through the CD28 CAR did not increase TCR/CD3ζ phosphorylation. However, there is a convergence in TCR and CAR downstream signaling, since the adaptor protein LAT, which is a linker between proximal and distal signaling events, becomes phosphorylated by each TCR and CAR activation, although at different levels (38).

**Figure 6:**
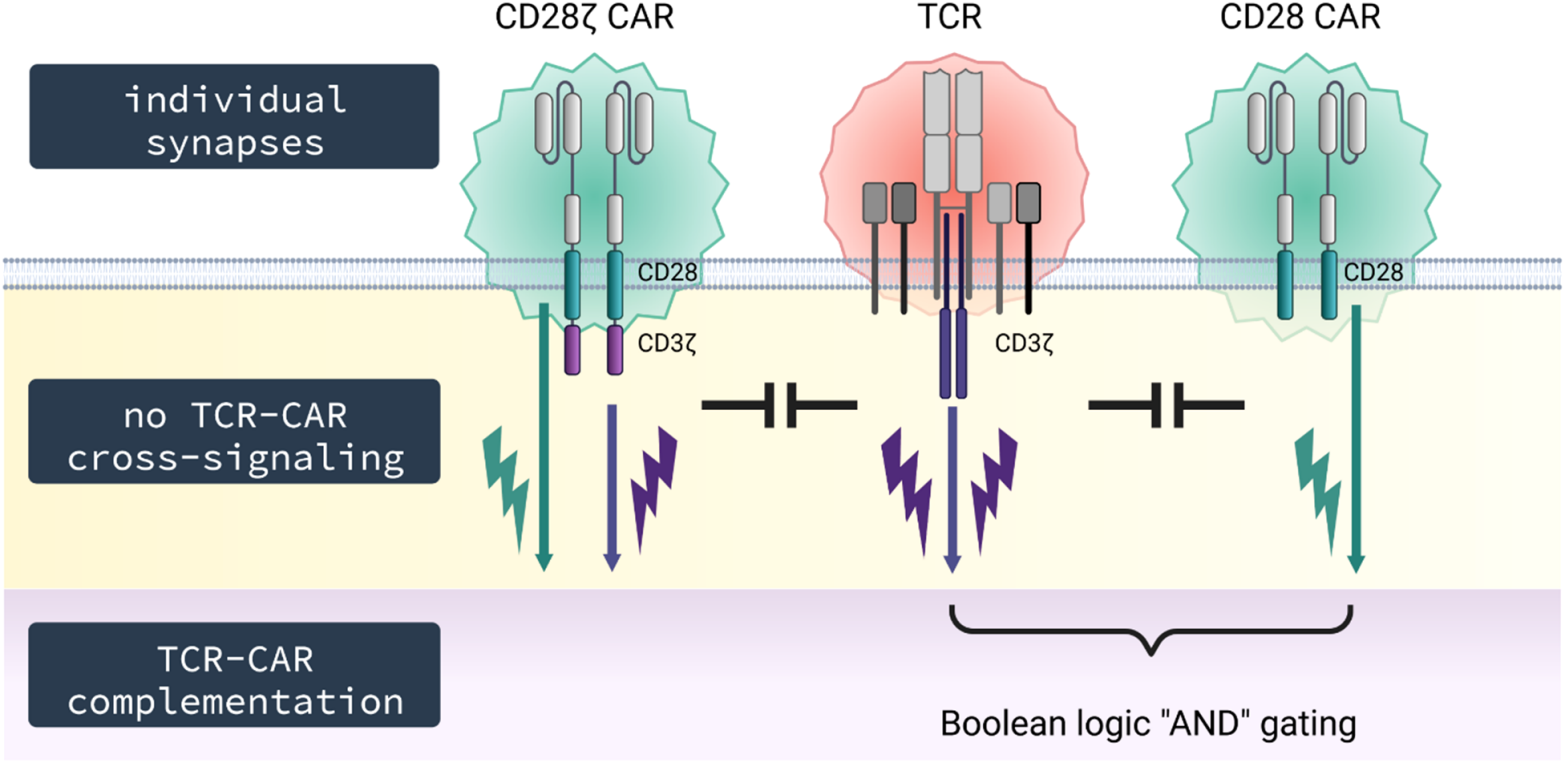
Schematic diagram. TCR and CAR do not co-integrate into the same synapse and signal independently upon engagement of their respective antigen without cross-signaling on the membrane level; however, TCR and CAR can complement in signaling upon simultaneous engagement of their respective cognate antigens, thereby providing T cell activation by both CD3 through the TCR and costimulation through the CAR.

We asked whether lack of cross-signaling at the early step was associated with lack of cross-activation of downstream pathways like the release of effector molecules including cytokines. To address this scenario in a well-defined antigen stimulation assay, we took advantage of the different signaling requirements for IFN−γ and IL−2 release; IFN−γ release indicates CD3ζ signaling while IL−2 release depends on combined CD3ζ and CD28 signaling in T cells. Using these cytokines as indicators, we revealed that signaling through TCR/CD3 did not activate CAR-associated CD28 and vice versa (**Figure 6**). However, TCR/CD3 stimulation can complement with CAR-provided CD28 co-stimulation when both TCR and CAR are engaging their respective cognate antigen; signaling through only the TCR or the CD28 CAR was not sufficient. Taken together data indicate lack of cross-signaling between CAR and TCR not only on the level of the cell membrane associated kinases but also in the downstream pathway of effector molecules. In addition to our findings, potential physical interaction between CAR and endogenous signaling molecules can occur. Muller et al. showed that CAR T cells harboring a CD28-derived transmembrane domain form heterodimers with the endogenous CD28; such CAR-CD28 heterodimers can activate CAR T cells (39). The number of molecules captured in heterodimers may differ and the functional consequences still need thorough investigation.

Our conclusions are of relevance for clinical applications in various aspects. Firstly, T cells will undergo terminal differentiation towards hypo-responsive cells with terminally differentiated KLRG-1^+^ CD57^+^ CD7^-^ phenotype once extensively stimulated through their TCR. In a previous study we revealed that hypo-responsiveness of CMV-specific late-stage CD8^+^ T cells is due to reduced TCR synapse formation compared to younger cells which is the result of galectin-mediated membrane-anchoring of TCR components (40). However, transgenic CAR expression and CAR triggering produced full effector functions in TCR hypo-responsive T cells indicating that the defect is restricted to TCR membrane components while synapse formation of the transgenic CAR was not blocked. CAR engineered late-stage T cells released cytokines and mediated redirected cytotoxicity as efficiently as younger effector T cells. Together with our recent analysis, data presented here sustain the model that CAR mediated activation occurs TCR-independently and can by-pass hypo-responsiveness of late-stage T cells upon repetitive TCR encounter.

Secondly, we do not expect an increase in signaling through the endogenous TCR in presence of a CAR, for instance, when EBV-specific T cells are used for a CAR redirected anti−tumor attack (41). Clinical observation indicates that both CAR and TCR can trigger T cells as TCR stimulation of virus-specific T cells in addition to CAR engagement of antigen enhances expansion of CAR T cells and finally their anti−leukemic function (42). Moreover, TCR and CAR can complement in signaling when simultaneously engaging their respective cognate antigen. This is of benefit when achieving complementation in target recognition; one target is recognized by the TCR, the other by the CD28 CAR as shown in our model system. Complementing in activation while lacking cross-signaling is the basis for creating Boolean logic “AND” gating by co-signaling through a CD28 CAR without primary signal while the latter is provided by signaling through the TCR upon engagement of its respective antigen. In this situation, only engagement of both targets will be capable to sustain a lasting T cell activation. The combination may also be used for specific T cell inhibition using an inhibitory CAR that dampens TCR driven activation upon CAR antigen recognition.

The concept of combinatorial antigen recognition was primarily introduced by Kloss et al. (43) aiming at complementing signals between two CARs, one CAR harboring a suboptimal activation signal and the other CAR harboring a costimulatory signal. So-called RevCARs are a further improvement as they represent an artificial receptor platform for controllable T cell activation (44). Herein, universal receptors are redirected by adaptor molecules to the respective targets allowing dosing of the adaptor molecules, flexible targeting and, notably in this context, combinatorial antigen recognition. Again, prerequisite for successful “AND” gating is lack of dimerization and cross-talk between the signaling receptors.

Thirdly, we do not expect altered CAR signaling under conditions where the endogenous CD3ζ chain is down-regulated as it occurs under chronic inflammatory conditions (45). CAR redirected T cell activation does not depend on primary TCR signals as it is mediated through the CAR intrinsic CD3ζ and costimulatory domain. However, the presence of the endogenous TCR/CD3 substantially prolongs the persistence of CAR T cells in a mouse model compared to TCR β−chain KO cells (11). This is the case despite similar CAR expression in both cells indicating the impact of the endogenous TCR/CD3 on sustaining CAR T cell function. The “tonic” activation through the TCR and thereby an active downstream signaling cascade, although at low levels, seems to be crucial for the overall therapeutic success given the less persistence of CAR redirected TCR KO T cells and the pivotal impact of CAR T cell persistence on their efficacy in controlling leukemia/lymphoma in the long-term. Along with this hypothesis, in patients treated with CAR engineered allogeneic TCR KO T cells only contaminating TCR^+^ CAR T cells, but not TCR^-^ CAR T cells, persisted while producing TCR signaling and finally graft-versus-host disease (9).

Taken together, CD3ζ CARs are similarly regulated as the CD3ζ chain of the TCR. However, the CAR cannot substitute for CD3ζ within the TCR complex underlining the concept that CAR and TCR form individual synapses in structure as verified by microscopic analyses and in function as shown by phospho-CD3ζ analyses. This specific situation allows logic “AND” gating by combinatorial target recognition through TCR and CAR. On the other hand, CAR engineered TCR KO T cells, designed for allogeneic “off-the-shelf” therapy, lack TCR support through “tonic” signaling and likely may lose functional capacities in the long-term.

## Data availability statement

The raw data supporting the conclusions of this article will be made available by the authors, without undue reservation.

## Author contributions

MB, AH, LV and MM-C conducted, designed, and analyzed experiments. AS, GV, AAH, GS, and HA interpreted the data and wrote the manuscript. All authors contributed to the article and approved the submitted version.

## Supplementary material

Supplementary Figure 1 Distribution of CAR, TCR and TfR in unstimulated and synapse forming cells. **(A)** Confocal and AiryScan Fast 2D fluorescence images of live anti-HER2 CAR T cells forming contacts with the tumor target. (B) Mean intensity of CAR-AF647, CAR-GFP, TCR-AF647, and TfR-AF647 in the synaptic region, extrasynaptic region, and both regions.

Supplementary Video 1 3D distribution of CAR-GFP and CAR-AF647 in the synaptic contact region of one exemplary cell. 3D fluorescence images of live anti-HER2 CAR T cells forming contacts with the tumor target were recorded in AiryScan Fast mode.

Supplementary Video 2 3D distribution of CAR-GFP and TCR-AF647 in the synaptic contact region and the extrasynaptic membrane of one exemplary cell. 3D fluorescence images of live anti-HER2 CAR T cells forming contacts with the tumor target were recorded in AiryScan Fast mode.

Supplementary Video 3 3D distribution of CAR-GFP and TfR-AF647 in the synaptic contact region and the extrasynaptic membrane of one exemplary cell. 3D fluorescence images of live anti-HER2 CAR T cells forming contacts with the tumor target were recorded in AiryScan Fast mode.

## References

[1] Porter DL Hwang W-T Frey NV Lacey SF Shaw PA Loren AW. Chimeric antigen receptor T cells persist and induce sustained remissions in relapsed refractory chronic lymphocytic leukemia. Sci Transl Med (2015) 7:303ra139. doi: 10.1126/scitranslmed.aac5415 PMC5909068 26333935

[2] Golumba-Nagy V Kuehle J Hombach AA Abken H. CD28-ζ CAR T cells resist TGF-β repression through IL-2 signaling, which can be mimicked by an engineered IL-7 autocrine loop. Mol Ther J Am Soc Gene Ther (2018) 26:2218–30. doi: 10.1016/j.ymthe.2018.07.005 PMC6127517 30055872

[3] Long AH Haso WM Shern JF Wanhainen KM Murgai M Ingaramo M. 4-1BB costimulation ameliorates T cell exhaustion induced by tonic signaling of chimeric antigen receptors. Nat Med (2015) 21:581–90. doi: 10.1038/nm.3838 PMC4458184 25939063

[4] Chandler NJ Call MJ Call ME. T Cell activation machinery: Form and function in natural and engineered immune receptors. Int J Mol Sci (2020) 21:7424. doi: 10.3390/ijms21197424 33050044 PMC7582382

[5] Hombach A Wieczarkowiecz A Marquardt T Heuser C Usai L Pohl C. Tumor-specific T cell activation by recombinant immunoreceptors: CD3 zeta signaling and CD28 costimulation are simultaneously required for efficient IL-2 secretion and can be integrated into one combined CD28/CD3 zeta signaling receptor molecule. J Immunol Baltim Md 1950 (2001) 167:6123–31. doi: 10.4049/jimmunol.167.11.6123 11714771

[6] Salter AI Ivey RG Kennedy JJ Voillet V Rajan A Alderman EJ. Phosphoproteomic analysis of chimeric antigen receptor signaling reveals kinetic and quantitative differences that affect cell function. Sci Signal (2018) 11:eaat6753. doi: 10.1126/scisignal.aat6753 30131370 PMC6186424

[7] Ramello MC Benzaïd I Kuenzi BM Lienlaf-Moreno M Kandell WM Santiago DN. An immunoproteomic approach to characterize the CAR interactome and signalosome. Sci Signal (2019) 12:eaap9777. doi: 10.1126/scisignal.aap9777 30755478 PMC6506216

[8] MacLeod DT Antony J Martin AJ Moser RJ Hekele A Wetzel KJ. Integration of a CD19 CAR into the TCR alpha chain locus streamlines production of allogeneic gene-edited CAR T cells. Mol Ther (2017) 25:949–61. doi: 10.1016/j.ymthe.2017.02.005 PMC5383629 28237835

[9] Qasim W Zhan H Samarasinghe S Adams S Amrolia P Stafford S. Molecular remission of infant b-ALL after infusion of universal TALEN gene-edited CAR T cells. Sci Transl Med (2017) 9:eaaj2013. doi: 10.1126/scitranslmed.aaj2013 28123068

[10] Ono S Ohno H Saito T. Rapid turnover of the CD3 zeta chain independent of the TCR-CD3 complex in normal T cells. Immunity (1995) 2:639–44. doi: 10.1016/1074-7613(95)90008-x 7796297

[11] Stenger D Stief TA Kaeuferle T Willier S Rataj F Schober K. Endogenous TCR promotes *in vivo* persistence of CD19-CAR-T cells compared to a CRISPR/Cas9-mediated TCR knockout CAR. Blood (2020) 136:1407–18. doi: 10.1182/blood.2020005185 PMC7612202 32483603

[12] Eshhar Z Waks T Gross G Schindler DG. Specific activation and targeting of cytotoxic lymphocytes through chimeric single chains consisting of antibody-binding domains and the gamma or zeta subunits of the immunoglobulin and T-cell receptors. Proc Natl Acad Sci U.S.A. (1993) 90:720–4. doi: 10.1073/pnas.90.2.720 PMC45737 8421711

[13] Rosskopf S Leitner J Paster W Morton LT Hagedoorn RS Steinberger P. A jurkat 76 based triple parameter reporter system to evaluate TCR functions and adoptive T cell strategies. Oncotarget (2018) 9:17608–19. doi: 10.18632/oncotarget.24807 PMC5915142 29707134

[14] Graham FL Smiley J Russell WC Nairn R. Characteristics of a human cell line transformed by DNA from human adenovirus type 5. J Gen Virol (1977) 36:59–74. doi: 10.1099/0022-1317-36-1-59 886304

[15] Hombach A Koch D Sircar R Heuser C Diehl V Kruis W. A chimeric receptor that selectively targets membrane-bound carcinoembryonic antigen (mCEA) in the presence of soluble CEA. Gene Ther (1999) 6:300–4. doi: 10.1038/sj.gt.3300813 10435115

[16] Heuser C Hombach A Lösch C Manista K Abken H. T-Cell activation by recombinant immunoreceptors: impact of the intracellular signalling domain on the stability of receptor expression and antigen-specific activation of grafted T cells. Gene Ther (2003) 10:1408–19. doi: 10.1038/sj.gt.3302023 12900755

[17] Hombach A Sircar R Heuser C Tillmann T Diehl V Kruis W. Chimeric anti-TAG72 receptors with immunoglobulin constant fc domains and gamma or zeta signalling chains. Int J Mol Med (1998) 2:99–103. doi: 10.3892/ijmm.2.1.99 9854151

[18] Schmidt P Kopecky C Hombach A Zigrino P Mauch C Abken H. Eradication of melanomas by targeted elimination of a minor subset of tumor cells. Proc Natl Acad Sci U.S.A. (2011) 108:2474–9. doi: 10.1073/pnas.1009069108 PMC3038763 21282657

[19] Chmielewski M Hombach A Heuser C Adams GP Abken H. T Cell activation by antibody-like immunoreceptors: Increase in affinity of the single-chain fragment domain above threshold does not increase T cell activation against antigen-positive target cells but decreases selectivity. J Immunol (2004) 173:7647–53. doi: 10.4049/jimmunol.173.12.7647 15585893

[20] Weijtens ME Willemsen RA Hart EH Bolhuis RL. A retroviral vector system “STITCH” in combination with an optimized single chain antibody chimeric receptor gene structure allows efficient gene transduction and expression in human T lymphocytes. Gene Ther (1998) 5:1195–203. doi: 10.1038/sj.gt.3300696 9930320

[21] Golumba-Nagy V Kuehle J Abken H. Genetic modification of T cells with chimeric antigen receptors: A laboratory manual. Hum Gene Ther Methods (2017) 28:302–9. doi: 10.1089/hgtb.2017.083 28741380

[22] Schrangl L. Sdt-python: Python library for fluorescence microscopy data analysis. (2020). doi: 10.5281/zenodo.4604495

[23] Harris CR Millman KJ van der Walt SJ Gommers R Virtanen P Cournapeau D. Array programming with NumPy. Nature (2020) 585:357–62. doi: 10.1038/s41586-020-2649-2 PMC7759461 32939066

[24] Virtanen P Gommers R Oliphant TE Haberland M Reddy T Cournapeau D. SciPy 1.0: fundamental algorithms for scientific computing in Python. Nat Methods (2020) 17:261–72. doi: 10.1038/s41592-019-0686-2 PMC7056644 32015543

[25] Huff J Bergter A Birkenbeil J Kleppe I Engelmann R Krzic U. The new 2D superresolution mode for ZEISS airyscan. Nat Methods (2017) 14:1223–3. doi: 10.1038/nmeth.f.404

[26] Schindelin J Arganda-Carreras I Frise E Kaynig V Longair M Pietzsch T. Fiji: an open-source platform for biological-image analysis. Nat Methods (2012) 9:676–82. doi: 10.1038/nmeth.2019 PMC3855844 22743772

[27] Ollion J Cochennec J Loll F Escudé C Boudier T. TANGO: a generic tool for high-throughput 3D image analysis for studying nuclear organization. Bioinformatics (2013) 29:1840–1. doi: 10.1093/bioinformatics/btt276 PMC3702251 23681123

[28] Jost LM Kirkwood JM Whiteside TL. Improved short- and long-term XTT-based colorimetric cellular cytotoxicity assay for melanoma and other tumor cells. J Immunol Methods (1992) 147:153–65. doi: 10.1016/s0022-1759(12)80003-2 1548398

[29] Li W Qiu S Chen J Jiang S Chen W Jiang J. Chimeric antigen receptor designed to prevent ubiquitination and downregulation showed durable antitumor efficacy. Immunity (2020) 53:456–470.e6. doi: 10.1016/j.immuni.2020.07.011 32758419

[30] Marin AV Jiménez-Reinoso A Briones AC Muñoz-Ruiz M Aydogmus C Pasick LJ. Primary T-cell immunodeficiency with functional revertant somatic mosaicism in CD247. J Allergy Clin Immunol (2017) 139:347–349.e8. doi: 10.1016/j.jaci.2016.06.020 27555457

[31] Blázquez-Moreno A Pérez-Portilla A Agúndez-Llaca M Dukovska D Valés-Gómez M Aydogmus C. Analysis of the recovery of CD247 expression in a PID patient: insights into the spontaneous repair of defective genes. Blood (2017) 130:1205–8. doi: 10.1182/blood-2017-01-762864 28743717

[32] Dombrowicz D Flamand V Miyajima I Ravetch JV Galli SJ Kinet JP. Absence of fc epsilonRI alpha chain results in upregulation of fc gammaRIII-dependent mast cell degranulation and anaphylaxis. evidence of competition between fc epsilonRI and fc gammaRIII for limiting amounts of FcR beta and gamma chains. J Clin Invest (1997) 99:915–25. doi: 10.1172/JCI119256 PMC507899 9062349

[33] Kraft S Wessendorf JH Hanau D Bieber T. Regulation of the high affinity receptor for IgE on human epidermal langerhans cells. J Immunol Baltim Md 1950 (1998) 161:1000–6. doi: 10.4049/jimmunol.161.2.1000 9670981

[34] Borkowski TA Jouvin MH Lin SY Kinet JP. Minimal requirements for IgE-mediated regulation of surface fc epsilon RI. J Immunol Baltim Md 1950 (2001) 167:1290–6. doi: 10.4049/jimmunol.167.3.1290 11466345

[35] van Vugt MJ Heijnen IA Capel PJ Park SY Ra C Saito T. FcR gamma-chain is essential for both surface expression and function of human fc gamma RI (CD64) in vivo. Blood (1996) 87:3593–9. doi: 10.1182/blood.V87.9.3593 8611682

[36] Roberts MR Cooke KS Tran AC Smith KA Lin WY Wang M. Antigen-specific cytolysis by neutrophils and NK cells expressing chimeric immune receptors bearing zeta or gamma signaling domains. J Immunol Baltim Md 1950 (1998) 161:375–84. doi: 10.4049/jimmunol.161.1.375 9647246

[37] Beppler C Eichorst J Marchuk K Cai E Castellanos CA Sriram V. Hyperstabilization of T cell microvilli contacts by chimeric antigen receptors. J Cell Biol (2023) 222:e202205118. doi: 10.1083/jcb.202205118 36520493 PMC9757849

[38] Salter AI Rajan A Kennedy JJ Ivey RG Shelby SA Leung I. Comparative analysis of TCR and CAR signaling informs CAR designs with superior antigen sensitivity and in vivo function. Sci Signal (2021) 14:eabe2606. doi: 10.1126/scisignal.abe2606 34429382 PMC8613804

[39] Muller YD Nguyen DP Ferreira LMR Ho P Raffin C Valencia RVB. The CD28-transmembrane domain mediates chimeric antigen receptor heterodimerization with CD28. Front Immunol (2021) 12:639818. doi: 10.3389/fimmu.2021.639818 33833759 PMC8021955

[40] Rappl G Riet T Awerkiew S Schmidt A Hombach AA Pfister H. The CD3-zeta chimeric antigen receptor overcomes TCR hypo-responsiveness of human terminal late-stage T cells. PLos One (2012) 7:e30713. doi: 10.1371/journal.pone.0030713 22292024 PMC3264628

[41] Savoldo B Rooney CM Di Stasi A Abken H Hombach A Foster AE. Epstein Barr Virus specific cytotoxic T lymphocytes expressing the anti-CD30zeta artificial chimeric T-cell receptor for immunotherapy of Hodgkin disease. Blood (2007) 110:2620–30. doi: 10.1182/blood-2006-11-059139 PMC1988944 17507664

[42] Lapteva N Gilbert M Diaconu I Al-Sabbagh M Rollins LA Naik S. T Cell receptor stimulation enhances the expansion and function of CD19 chimeric antigen receptor-expressing T cells. Clin Cancer Res (2019) 25:7340–7350. doi: 10.1158/1078-0432.CCR-18-3199 PMC7062259 31558475

[43] Kloss CC Condomines M Cartellieri M Bachmann M Sadelain M. Combinatorial antigen recognition with balanced signaling promotes selective tumor eradication by engineered T cells. Nat Biotechnol (2013) 31:71–5. doi: 10.1038/nbt.2459 PMC5505184 23242161

[44] Feldmann A Hoffmann A Kittel-Boselli E Bergmann R Koristka S Berndt N. A novel revcar platform for switchable and gated tumor targeting. Blood (2019) 134:5611. doi: 10.1182/blood-2019-128436

[45] Berg L Rönnelid J Klareskog L Bucht A. Down-regulation of the T cell receptor CD3 zeta chain in rheumatoid arthritis (RA) and its influence on T cell responsiveness. Clin Exp Immunol (2000) 120:174–82. doi: 10.1046/j.1365-2249.2000.01180.x PMC1905626 10759780

