# Supplementary figures and images for: CAR and TCR form individual signaling synapses and do not cross-activate, however, can co-operate in T cell activation
Source: Front Immunol. 2023 Feb 1;14:1110482. doi: 10.3389/fimmu.2023.1110482 (PMC9929185; doi:10.3389/fimmu.2023.1110482)

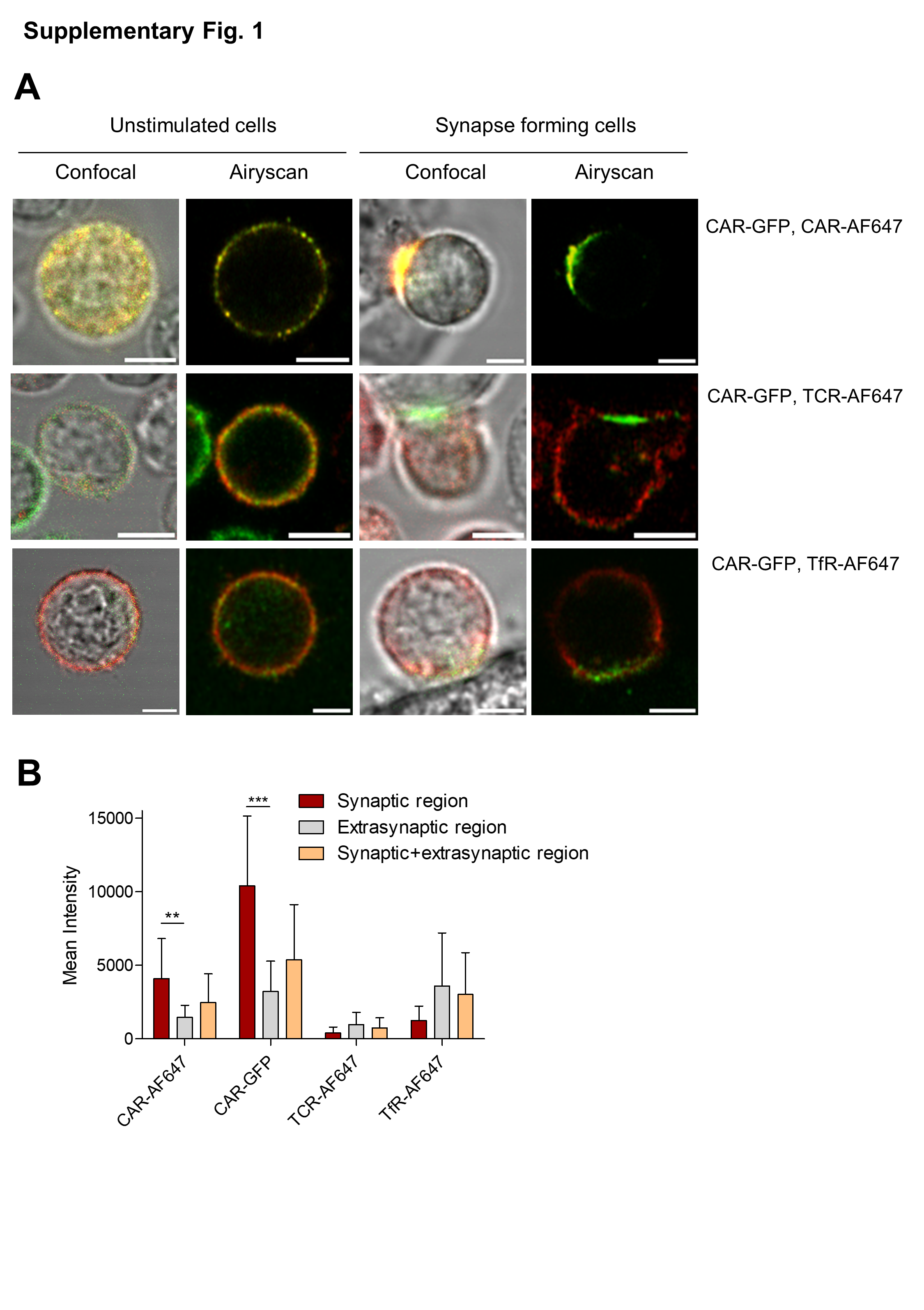

Supplement: Supplementary Figure 1 — Distribution of CAR, TCR and TfR in unstimulated and synapse forming cells. (A) Confocal and AiryScan Fast 2D fluorescence images of live anti-HER2 CAR T cells forming contacts with the tumor target. (B) Mean intensity of CAR-AF647, CAR-GFP, TCR-AF647, and TfR-AF647 in the synaptic region, extrasynaptic region, and both regions. [file Image_1.tif]
